# Network pharmacology analysis of Lanatoside C: molecular targets and mechanisms in the treatment of ulcerative colitis

**DOI:** 10.3389/fmolb.2025.1552360

**Published:** 2025-03-21

**Authors:** Wenjing Zhu, Zhengjie Zhang, Xinyuan Wang

**Affiliations:** ^1^ College of Art, Jiangsu Open University, Nanjing, China; ^2^ School of Biochemistry and Immunology, Trinity Biomedical Sciences Institute, Trinity College Dublin, Dublin, Ireland

**Keywords:** colitis, Lanatoside C, network pharmacology, molecular docking, experimental verification

## Abstract

**Introduction:**

Ulcerative colitis (UC) is a chronic and progressive inflammatory disease of the intestines, marked by recurrent inflammation along the digestive tract, leading to symptoms such as bloody diarrhea and weight loss, severely impacting patients’ quality of life. Despite extensive research, current therapeutic treatment for UC still faces challenges in long-term efficacy and safety. Lanatoside C (LanC), as a type of cardiac glycosides, has shown promising anti-inflammatory effects. This study employs network pharmacology to investigate the effects and mechanisms of LanC in the treatment of UC.

**Method:**

LanC- and UC-associated target genes datasets were retrieved from the Genecards, DisGeNET, and Gene Expression Omnibus database. Integration analysis identified a common set of potential LanC targets for UC treatment. Analyses of Gene ontology (GO) and Kyoto Encyclopedia of Genes and Genomes (KEGG) were performed on these target genes. Additionally, a protein-protein interaction (PPI) network was constructed to identify the top targets with the highest connectivity. Molecular docking and cellular experiments were subsequently carried out to further validated these findings.

**Results:**

23 intersecting genes were identified as potential targets of LanC in UC. Among these, KDR, STAT3, ABCB1, CYP3A5, and CYP2B6 emerged as the top 5 targets with high therapeutic potential. Pathway analysis indicated the involvement of fatty acid and lipid metabolism, as well as xenobiotic metabolism pathways, which could be crucial for LanC′s efficacy in treating UC. Molecular docking simulations revealed favorable binding interaction between LanC and KDR, STAT3, ABCB1, CYP3A5, and CYP2B6. Furthermore, *In vitro* experiments demonstrated that LanC significantly inhibits LPS-induced pro-inflammatory cytokines expression in RAW264.7 cells.

**Conclusion:**

This study demonstrates a comprehensive overview of the therapeutic potential of LanC in UC and elucidates its mechanisms of action. These findings offer a theoretical basis for further optimizing UC clinical therapy and underscore the potential of LanC as a novel therapeutic option for UC.

## 1 Introduction

Ulcerative colitis (UC), a predominant form of inflammatory bowel disease (IBD), is characterized by chronic, recurrent inflammation of the digestive tract (Gajendran et al., 2019). The prolonged disease course and high recurrence rate significantly impair patients’ quality of life (Armuzzi and Liguori, 2021). More critically, one of the most severe and life-threatening complications of UC is the development of colitis-associated colorectal cancer (CAC) (Chen et al., 2024). Current therapeutic strategies for UC include 5-aminosalicylic acid medications, glucocorticoids, immunosuppressants, and biological agents (Neurath, 2017; Aslam et al., 2022). However, these treatments often limited by concerns related to long-term efficacy and safety (Aslam et al., 2022), Therefore, the development of novel, effective, and safer therapeutic agents for UC remains an urgent priority. In recent years, accumulating evidence has highlighted the therapeutic potential of natural compounds in UC management. For instance, Gingerenone A, derived from *Zingiber officinale*, has been shown to directly bind to IL-17RA and inhibit the activation of inflammatory signaling pathways, thereby preventing and treating UC (Liang et al., 2024). Similarly, Puerarin, an active compound extracted from Pueraria lobata, has been found to suppress M1 macrophage polarization *in vitro*, which is sufficient to exert therapeutic effects on colonic lesions and systemic inflammation in DSS-induced colitis mouse models (Tao et al., 2024). These studies highlighting the need for further exploration of natural compounds as potential therapeutic adjuncts for UC.

Cardiac glycosides comprise a diverse family of naturally occurring compounds characterized by a shared structural motif (Botelho et al., 2019). The steroidal structure at the core of their molecular framework serves as the pharmacophoric moiety responsible for their biological activity, primarily modulating cation homeostasis in mammalian cells by inhibiting Na^+^/K^+^ ATPase function (Bejcek et al., 2021). This mechanism significantly impacts cardiomyocytes, influencing heart rate and contractility, making cardiac glycosides crucial in clinical management of heart failure (Botelho et al., 2019). Additionally, extensive research has revealed their potential in anti-tumor (Cheng et al., 2019; Ayogu and Odoh, 2020; Kumavath et al., 2021), anti-inflammatory (Jansson et al., 2021; Skubnik et al., 2021; Olona et al., 2022), and anti-viral activities (Reddy et al., 2020; Ouyang et al., 2024). Among these compounds, Lanatoside C (LanC), an FDA-approved cardiac glycoside, is widely utilized in the treatment of heart failure and cardiac arrhythmia. Recent studies have shown its anti-inflammatory potential, such as modulating neuroinflammation and exhibiting protective effects in pulmonary fibrosis by suppressing fibroblast proliferation and differentiation (Jansson et al., 2021). Additionally, its protective effects have been observed in a murine model of Bleomycin-induced pulmonary fibrosis, attributed to the suppression of fibroblast proliferation and differentiation (Nie et al., 2019). Despite these promising findings, there is a notable absence of research on LanC’s therapeutic effects, target genes, and mechanisms in UC. Investigating these aspects may present a novel approach to advancing UC treatment strategies.

Network pharmacology, an interdisciplinary field integrating high-throughput histology, bioinformatics, and systems biology, plays a crucial role in understanding complex biological systems (Hopkins, 2008). This methodology enables the identification of drug targets, exploration of drug-disease interactions, and mapping of therapeutic pathways, thereby offering valuable insights into drug mechanisms (Wu et al., 2022; Zhang et al., 2022). In this study, we employed network pharmacology, combined with docking analysis and *In vitro* validation to investigate the potential pharmacological mechanisms of LanC in UC.

## 2 Materials and methods

### 2.1 Network pharmacology analysis of LanC in the treatment of UC

#### 2.1.1 Collection of differentially expressed genes in UC

Transcriptional profile data of ulcerative colitis were obtained from two datasets with the keywords ‘Ulcerative colitis’: GSE87466 (108 samples, including 21 normal and 87 UC samples) and GSE222070 (27 samples, including 10 normal and 17 UC samples) from the Gene Expression Omnibus database (GEO, https://www.ncbi.nlm.nih.gov/geo/). Separate analyses were performed for each dataset by using the limma package, applying criteria of *p-value* < 0.05 and |logFC| > 0.5.

#### 2.1.2 Target prediction of LanC

The SMILES chemical structure of LanC was retrieved from the PubChem database (https://pubchem.ncbi.nlm.nih.gov/). This structure was utilized as input in the chEMBL (https://www.ebi.ac.uk/chembl/), Super-PRED (https://prediction.charite.de/), and DGIdb (https://www.dgidb.org/) databases to predict target genes. Upon integration of the data and the removal of duplicated target genes, a comprehensive set of LanC-associated target genes was established.

#### 2.1.3 Collection of UC-related therapeutic target genes

Therapeutic target genes of UC were collected from Genecards (https://www.genecards.org/) and DisGeNET (https://www.disgenet.org/) databases using the keywords ”ulcerative colitis”. Upon integration of the data and the removal of duplicated target genes, a comprehensive set of target genes associated with UC was established.

#### 2.1.4 Prediction of LanC target genes in UC

The integration of 768 LanC-associated target genes, 5687 UC-related genes, and differentially expressed genes, comprising 3,608 from the GSE87466 dataset and 7,401 from the GSE222070 dataset, a subset of 23 genes were found to be shared among these datasets and delineated with the Venn diagram plotted by the R language Venn Diagram package for intuitive vision.

#### 2.1.5 Function and pathway enrichment analysis

The R software (version 4.2.0) was utilized to install “colorspace,” “stringi,” and “ggplot2” packages. The Bioconductor package, encompassing “DOSE,” “clusterProfiler,” and “enrichplot,” was applied to perform Gene Ontology (GO) and Kyoto Encyclopedia of Genes and Genomes (KEGG) enrichment analyses on the identified 23 intersecting target genes. The “enrichGO” function was employed for Gene Ontology (GO) enrichment analysis, with parameters set as OrgDb = “org.Hs.eg.db,” keyType = “ENTREZID,” and ont = “ALL.” Additionally, the “enrichKEGG” function was utilized for the Kyoto Encyclopedia of Genes and Genomes (KEGG) enrichment analysis, with parameters set as organism = “hsa” and keyType = “kegg.” The filter of the *P value* for both functions was set to 0.05. The top 10 enrichment results from GO enrichment and KEGG enrichment were visualized as a point graph and a bar plot graph, respectively.

#### 2.1.6 Conducting protein-protein interaction (PPI) network

The 23 intersecting target genes were analyzed for PPI network using the STRING database (https://cn.string-db.org/). The PPI network was visualized through graphical representation using Cyto-scape software (version 3.9.1). Concurrently, the Cytohubba plugin was employed, utilizing the Maximum Clique Centrality (MCC) algorithm to identify the top 5 core target proteins. The Layout section was configured with the Degree Sorted Circle layout.

#### 2.1.7 Top 5 LanC target genes-pathways network construction

The software Cytoscape3.0.1 was employed to create a network illustrating the relationships between LanC, its target genes, and UC-related pathways.

#### 2.1.8 Molecular docking

The protein structures of ABCB1, CYP3A5, KDR, CYP2B6, and STAT3 were downloaded from the RCSB Protein Data Bank (http://www.pdb.org/). The Isomeric SMILES sequence of LanC was obtained from the PubChem database (https://pubchem.ncbi.nlm.nih.gov/) and converted to a 3D model structure with lowest energy by using NavoPro online platform (https://www.novopro.cn/tools/smiles2pdb) through the MM2 module. The 3D model structure of LanC was processed in AutoDockTools-1.57 software to adjust charges, identify ligand roots, and select rotatable ligand-bonding sites. The final output was saved as a pdbqt format file. Meanwhile, the pdb files of the target proteins were input into PyMOL software for pre-processing to remove water molecules. Subsequently, the pre-processed protein pdb files were imported into AutoDockTools-1.5.7 software to process proteins as follows: remove the native ligand, add non-polar hydrogens, optimize amino acids, and calculate the Gasteiger charges. The active binding site within the protein was determined based on the original ligand’s position. Molecular docking simulations and binding energy determinations were conducted using AutoDock Vina software by inputting parameters such as receptor name, ligand name, docking center coordinates, and distance. The partial diagrams of molecular docking were generated using the PyMOL software.

### 2.2 Cellular experiments

#### 2.2.1 Cell culture

RAW264.7 murine macrophage cells were obtained from ATCC and cultured in high-glucose Dulbecco’s modified Eagle’s medium (DMEM) (Gibco) with 10% (v/v) fetal bovine serum (FBS) (Gibco), 100 μg/mL Penicillin and 100 μg/mL Streptomycin (NCM Biotech). Cells were cultured in a 37°C humidified chamber under a 5% CO_2_ atmosphere.

#### 2.2.2 Screening of cellular drug delivery concentrations

RAW264.7 cells were seeded at a density of 5,000 cells per well in 96-well plates. Following 24 h incubation, cells were treated with indicated concentrations of LanC (MedChemExpress) for 24 h. CCK-8 solution (MedChemExpress) was added to the wells and the plates were returned to the 37°C humidified chamber under a 5% CO_2_ atmosphere for 1 h. The absorbance (A) values of cells were assessed at 450 nm using an enzyme marker, and the cell viability was computed. Cell viability (%) = (A sample − A blank)/(A control − A blank)* 100%. The half maximal inhibitory concentration (IC50) of LanC to the cell viability was determined by interpolation from dose-response curves.

#### 2.2.3 Enzyme-linked immunosorbent (ELISA) assay

RAW264.7 cells were seeded at a density of 1 × 10^6^ cells per well in 96-well plates. Following 24 h incubation, cells were pre-treated with the indicated concentration of LanC for 1 h and then stimulated with 1 μg/mL LPS (Beyotime) for 24 h. Conditional media was collected and IL-1β (MLB00C), IL-6 (M6000B), and TNF-α (MTA00B) were quantified by sandwich ELISA kits from R&D Systems.

#### 2.2.4 Quantitative reverse transcription-PCR

RAW264.7 cells were seeded at a density of 5 × 10^6^ cells per well in 6-well plates. Following 24 h incubation, cells were pre-treated with the indicated concentration of LanC for 1 h and then stimulated with 1 μg/mL LPS for 24 h. Total RNA was isolated from the cells using the Total RNA Isolation Kit (Omegabiotek). Reverse transcription was performed using the Prime Script TM RT-PCR kit (Takara), and RT-PCR was carried out using the Biorad CFX Connect (Biorad), as per the manufacturer’s instructions. The relative expression of each target gene mRNA were normalized to the housekeeping gene Hypoxanthine-guanine phosphoribosyltransferase (Hprt) by using the 2^−ΔΔCT^ method. The following primers were used to amplify the indicated genes in this study were synthesized by GenScript Biotech: Mouse *IL-1β*, forward, ATGCCACCTTTTGACAGTGATG, and reverse, TGATGTGCTGCTGCGAGATT; Mouse *IL-6*, forward, TAGTCCTTCCTACCCCAATTTCC, and reverse, TTGGTCCTTAGCCACTCCTTC; Mouse *TNFα*, forward, CCTGTAGCCCACGTCGTAG, and reverse, GGGAGTAGACAAGGTACAACCC; Mouse *STAT3*, forward, CAATACCATTGACCTGCCGAT, and reverse, GAGCGACTCAAACTGCCCT; Mouse *KDR*, forward, GAAACAGGTGAGGTAGGCAG, and reverse, ACCCTCGTTTTCAGAGTTGG; Mouse *ABCB1a*, forward, TCCTCACCAAGCGACTCCGATA, and reverse, ACTTGAGCAGCATCGTTGGCGA; Mouse *ABCB1b*, forward, GGTGGTGTCATTGTGGAGCAAG, GCATCAGTGTCACTCTGGGATC; *Hprt*, forward, GTCCCAGCGTCGTGATTAGC, and reverse, TGGCCTCCCATCTCCTTCA.

### 2.3 Statistical analysis

The data were presented as mean ± SD, and each assay was conducted in triplicate under identical experimental conditions. Permutation test and Bonferroni correction were employed to assess intergroup differences, with statistical significance set at *p-value* < 0.05.

## 3 Results

### 3.1 Screening for differentially expressed genes in UC

**FIGURE 1:**
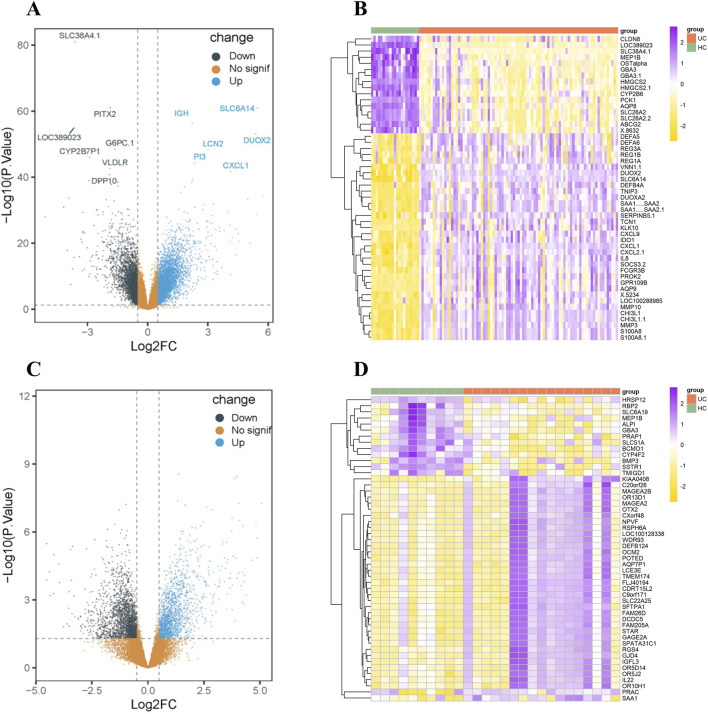
Gene Expression Analysis of differentially expressed genes in UC from the GSE87466 and GSE222070 datasets. **(A)** Gene volcano map of 7,401 genes exhibiting altered expression levels in the GSE87466 dataset. **(B)** Gene heat map of top 50 differentially expressed genes in the GSE87466 dataset. **(C)** Gene volcano map of 4,320 genes exhibiting altered expression levels in the GSE222070 dataset. **(D)** Gene heat map of top 50 differentially expressed genes in the GSE222070 dataset.

To identify genes with altered expression in UC, two publicly datasets, GSE87466 and GSE222070, were acquired from the Gene Expression Omnibus (GEO) database. Within these datasets, specimens from UC patients and healthy controls (HC) were selected for analysis. After employing the limma package, a total of 7,401 genes with modified expression levels were identified in the GSE87466 dataset, with 4,320 upregulated and 3,081 downregulated genes displayed in UC compared to HC samples, with a significance threshold of -log *P* > 40 (Figure 1A). The top 50 differentially expressed genes, ranked by the highest absolute log_2_ (fold change), are visually represented in the gene heat map (Figure 1B; Supplementary Table S1). Gene expression levels are visually represented using a colors gradient: yellow indicates a decrease, purple represents an increase, and the brightness of color reflects the degree of gene expression alteration. Similarly, in the GSE222070 dataset, a total of 3,608 genes showed altered expression profiles, with 1,590 upregulated and 2018 downregulated genes. Gene volcano plots (Figure 1C) and gene heat maps (Figure 1D; Supplementary Table S2) were constructed using parallel methodologies.

### 3.2 Target genes screening of LanC in UC

We next utilized the SMILES chemical structure of LanC from the PubChem database. Computational predictions for target genes were conducted through the Super-PRED database, resulting in 124 potential target genes being identified. Additionally, we extended our queries in the chEMBL, Genecards (Relevance score ≥1) and DGIdb databases to identify 612, 52 and 15 target genes associated with LanC, respectively. After integrating these datasets and removing duplicates, we compiled a comprehensive list of 768 target genes. Target genes associated with UC were retrieved from the Genecards (Relevance score ≥1) and DisGeNET databases by using the keyword “Ulcerative Colitis”, which includes 5,332 genes in Genecards and 1,482 genes in DisGeNET, respectively. After consolidating the data and removing duplicate gene names, a total of 5,687 target gene datasets were compiled.

**FIGURE 2:**
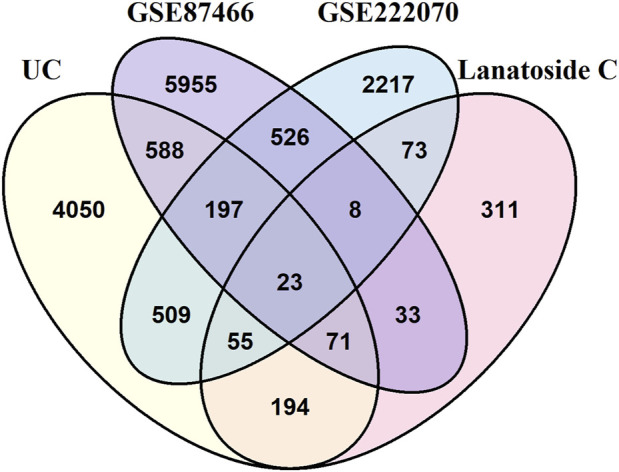
The intersection of identified target genes of LanC and UC.

Subsequently, intersection analysis was performed using *VennDiagram* to identify shared genes among the 5687 UC-related genes, the 768 LanC target genes, the 7401 UC-related differentially expressed genes from the GSE87466 transcriptome dataset, and the 3608 UC-related differentially expressed genes from the GSE222070 transcriptome dataset. This analysis revealed 23 overlapping genes consistently present across all datasets, which were designated as key targets for further investigation (Figure 2; Table 1).

**TABLE 1:** 23 overlapping target genes of LanC and UC.

geneName	geneName	geneName
STAT3	KDR	KCNA3
ABCB1	GUCY2C	UGT2B17
CCR6	CD74	CDK18
PPARG	FGFR2	MALT1
PRKCB	NLRP1	PRKD1
CCR5	BLK	CYP2J2
HPGDS	CYP2B6	CYC1
CYP3A5	FAAH	

### 3.3 Analysis of GO function and KEGG enrichment analysis of 23 overlapping target genes

**FIGURE 3:**
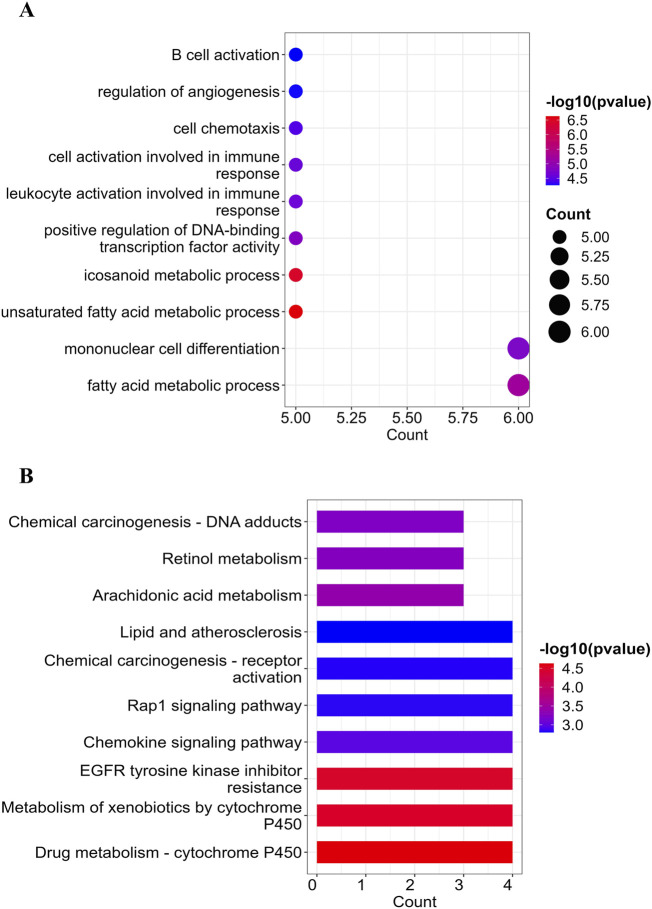
GO and KEGG enrichment analysis of the 23 overlapping target genes. **(A)** the point map of GO enrichment analysis of the LanC-UC 23 overlapping target genes, including the top 10 enrichment terms of BP. **(B)** the bar point map of KEGG enrichment analysis of the LanC-UC 23 overlapping target genes (top 10).

Following the identification of 23 overlapping target genes associated with both LanC and UC, Gene Ontology (GO) and Kyoto Encyclopedia of Genes and Genomes (KEGG) pathway enrichment analysis were performed to investigate the biological roles and related pathways. GO enrichment analysis (*p* < 0.05 and *q* < 0.05) categorized the target genes into three major domains: Biological Process (BP), Molecular Function (MF), and Cellular Component (CC). In total, 214 GO terms were enriched, comprising 165 BP terms, 47 MF terms, and 2 CC terms (Supplementary Table S3). The top 10 BP terms with the lowest *P value* are visualized in a dot plot (Figure 3A), highlighting key processes such as fatty acid metabolism, xenobiotic metabolism, transcription factor activity regulation, monocyte differentiation, and immune cell activation. For KEGG pathway enrichment analysis (*p-value* < 0.05 and *q* < 0.05), 13 signaling pathways were identified (Supplementary Table S4). The top 10 signaling pathways with the lowest *P value* are illustrated in a bar plot (Figure 3B), and include pathways related to cell metabolism, signaling pathway activation, resistance to epidermal growth factor receptor (EGFR) tyrosine kinase inhibitors, and lipid and atherosclerosis processes.

### 3.4 Construction of LanC-UC-related PPI network

**FIGURE 4:**
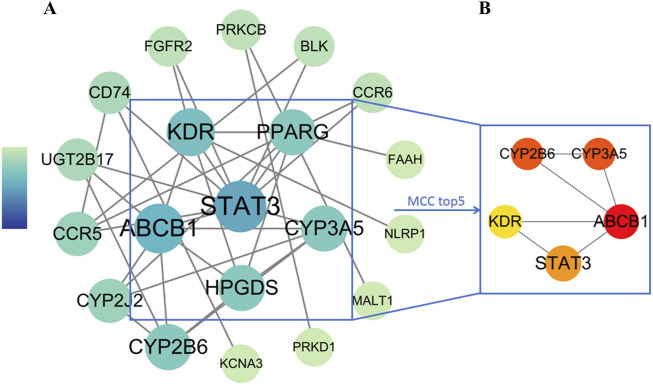
PPI network analysis of LanC-UC-related target genes. **(A)** The visualization of the PPI network for 23 overlapping target genes acquired from the STRING database through Cytoscape 3.9.1. The degree connectivity metric was adjusted both with size and color from green (low) to blue (high). **(B)** The top 5 core proteins were revealed by the MCC algorithm. The degree value was measured by color from yellow (low) to red (high).

To understand the interactions among the 23 overlapping target genes of LanC in the context of UC, we used the STRING database and performed computational analysis in Cytoscape 3.9.1 with the Analyze Network tool. Node size and color were adjusted based on degree connectivity metric, transitioning from green (low) to blue (high). This resulted in a protein-protein interaction (PPI) network comprising 20 protein nodes and 34 edges (Figure 4A). Furthermore, Using the Cytohubba plugin with the Maximum Neighborhood Component (MCC) algorithm, we identified the top 5 core proteins: KDR (VEGFR2), STAT3, ABCB1, CYP3A5, and CYP2B6 (Figure 4B).

### 3.5 LanC-top 5 target genes-pathways network

**FIGURE 5:**
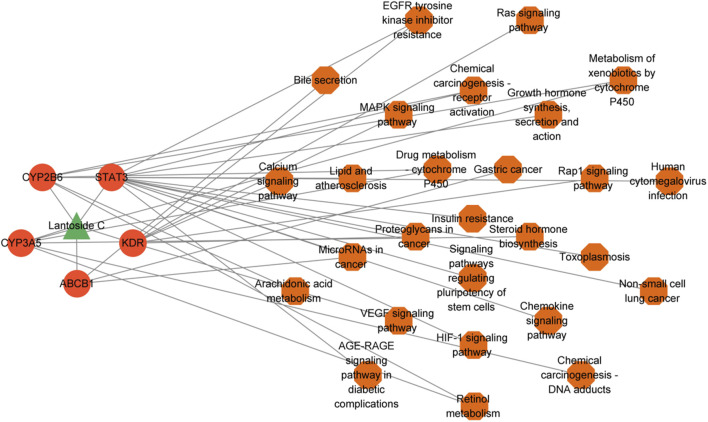
The construction of LanC-target gene-pathway network. The green triangle represents LanC, the red-orange circles represent the top 5 target proteins, and the orange hexagon represents the correlated signaling pathways. The gray lines represent their interaction.

LanC-target gene-pathway network analysis was established to demonstrate the relationship between LanC, potential target genes and the correlated pathways. As shown in Figure 5, the LanC-target gene-pathway interaction network works with 33 nodes and 44 edges.

### 3.6 Molecular docking models of LanC with top 5 target proteins

**FIGURE 6:**
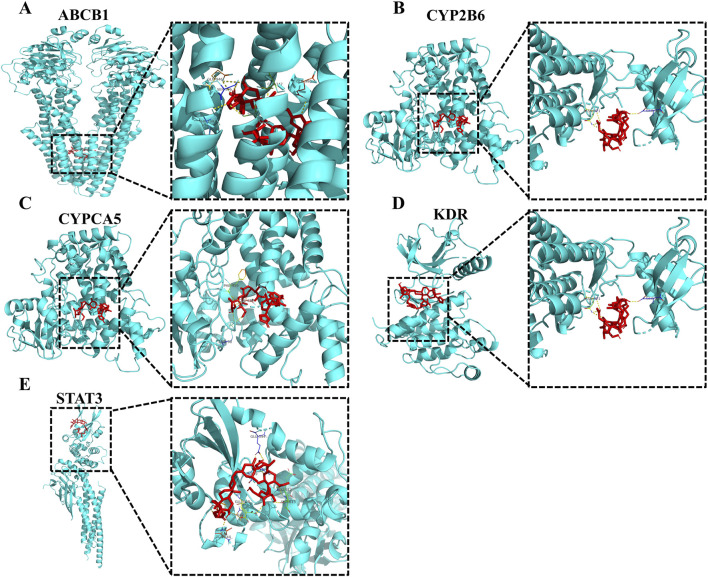
Partial diagram of molecular docking of LanC with top 5 target proteins. **(A)** LanC-ABCB1. **(B)** LanC-CYP2B6. **(C)** LanC-CYPCA5. **(D)** LanC-KDR. **(E)** LanC-STAT3.

To further confirm the effects of LanC on the top 5 core target proteins (KDR, STAT3, ABCB1, CYP3A5, and CYP2B6), we performed molecular docking to investigate the possibility of their interactions. This technique simulates the interaction between small ligand molecules and receptor protein macromolecules, with binding energy calculated to predict affinity. Binding energies less than 0 kcal/mol indicate spontaneous combination and greater stability with smaller energies. The two-dimensional structure of LanC was downloaded from the PubChem database for molecular docking. The predictive results indicate that LanC exhibits favorable binding with five target proteins (Figure 6), and the binding energies are consistently below 0 kcal/mol (Table 2).

**TABLE 2:** Molecular docking of LanC binding energy with top5 target proteins.

Target	PDB	Pubchem_ID	Compound	Free binding energy (kcal/mol)
ABCB1	7a69	656,630	LanC	−10.65
CYP2B6	3ibd			−7.427
CYPCA5	7lad			−6.85
KDR	1y6a			−9.025
STAT3	6njs			−5.024

### 3.7 Anti-inflammatory effect of LanC in RAW264.7 cell

**FIGURE 7:**
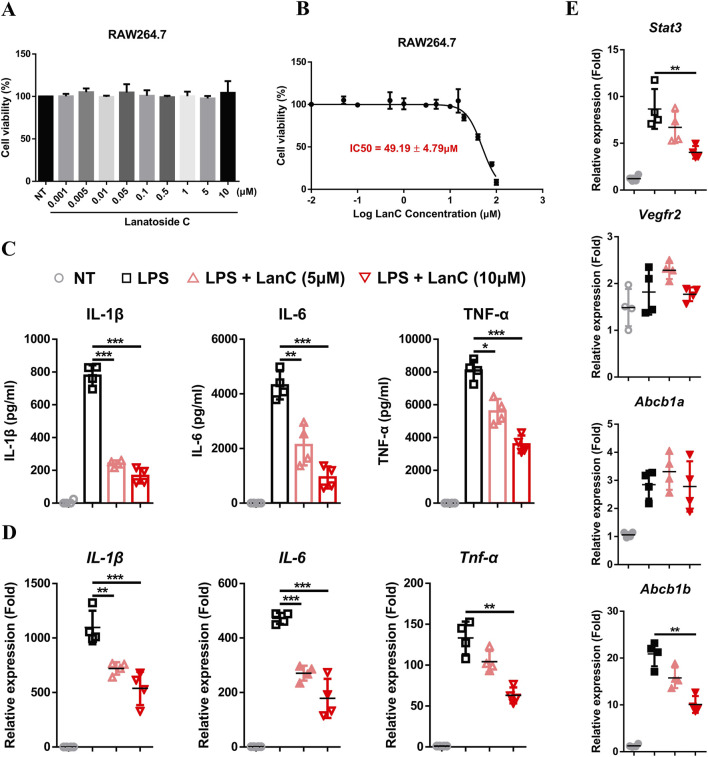
Anti-inflammatory effects of LanC in RAW264.7 cells. **(A)** RAW264.7 cells were treated with indicated concentrations of LanC for 24 h and cell viabilities were detected by CCK-8 assay. **(B)** RAW264.7 cells were treated with indicated concentrations of LanC for 24 h and IC50 of LanC were calculated with cell viability through CCK-8 assay. **(C)** RAW264.7 cells were pre-treated with 5 μM or 10 μM LanC for 1 h and stimulated with 1 μg/mL LPS for 24 h, supernatants were assayed by ELISA for levels of IL-1β, IL-6 and TNF-α. **(D, E)** RAW264.7 cells were pre-treated with 5 μM or 10 μM LanC for 1 h and stimulated with 1 μg/mL LPS for 24 h, mRNA were assayed by Real-time PCR for levels of IL-1β, IL-6, and TNF-α **(D)** or STAT3, KDR, ABCB1a, and ABCB1b **(E)**. Data were collected from three independent experiments and presented as means ± SD. All data statistical differences were evaluated using Permutation test and Bonferroni correction. *p < 0.05, **p < 0.01, ***p < 0.001.

To validate the efficacy of LanC in treating UC, as suggested by network pharmacology assays, we decided to conduct cellular experiments. The cytotoxicity of LanC on RAW264.7 cell viability was assessed using the CCK-8 assay. As described in Figure 7A, LanC at concentrations of 5 μM and 10 μM showed no significant detrimental effects on RAW264.7 cell viability. Further calculation of the IC50 revealed that LanC exerted an IC50 of 49.19 ± 4.79 μM on RAW264.7 cells (Figure 7B). Therefore, concentrations of 5 μM and 10 μM were selected for subsequent experiments as they did not induce toxicity to RAW264.7 cells.

To investigate the anti-inflammatory effects of LanC, RAW246.7 cells were stimulated with LPS. Compared to the LPS control group, LanC significantly reduced the LPS-induced expression of pro-inflammatory cytokines IL-1β, IL-6, and TNF-α (Figure 7C) in a notable dose-dependent manner. Real-time PCR results further confirmed that LanC effectively suppressed the LPS-induced mRNA transcription of these three cytokines (Figure 7D), with this inhibitory effect similarly demonstrating a dose-dependent trend. Subsequently, to further elucidate the underlying mechanism of LanC’s anti-inflammatory effects, we examined the mRNA expression levels of five identified core target genes that were previously described (Figure 4). Literature review indicated that the expression of STAT3 (Han and Theiss, 2014; Huang et al., 2022), KDR (Knod et al., 2013; Alkim et al., 2015), and ABCB1 (Djouina et al., 2022) are closely related to UC occurrence and development. Thus, we decided to focus on these three target genes. Results showed that LPS failed to induce an upregulation in the mRNA expression levels of VEGFR2, but significantly activated the transcription of STAT3, ABCB1a, and ABCB1b. Meanwhile, LanC inhibited the mRNA expression level of STAT3 and ABCB1b (Figure 7E).

## 4 Discussion

UC is an inflammatory disorder of the colonic mucosa with an unknown etiology, given its unclear pathogenesis and potential insidiousness, the therapeutic objectives for UC have shifted from only alleviating symptoms to achieving mucosal repair. However, conventional treatments are mainly limited to managing inflammation and clinical manifestations, failing to meet long-term treatment needs (Kobayashi et al., 2020), and acute enteritis is prone to transform into chronic enteritis, which in turn develops into colorectal cancer (CAC) (Xu et al., 2024). Therefore, exploring novel therapeutic targets is urgently needed for the management of UC.

This study initially explored the potential and mechanisms of LanC for the treatment of UC based on network pharmacology. Our results indicated that the therapeutic mechanism of LanC in UC is complex, involving multiple protein targets and pathways. Through Gene Ontology (GO) and Kyoto Encyclopedia of Genes and Genomes (KEGG) pathway analyses, key pathways of LanC in treating UC were identified, including fatty acid metabolism, cytochrome metabolism, cell activation immune response, and positive regulation of transcription factors. Subsequently, through PPI network topology screening, CYP3A5, CYP2B6, STAT3, KDR, and ABCB1 were identified as the top 5 major potential core targets. Furthermore, molecular docking demonstrated excellent binding interactions between LanC molecules and these five targets, as indicated by binding affinity and hydrogen bond results.

Cellular experiments with RAW264.7 murine macrophages demonstrated that LanC effectively inhibits the LPS-induced expression of pro-inflammatory cytokines IL-1β, IL-6, and TNF-α on both protein and mRNA levels. Further literature review on the core targets and UC revealed that STAT3, KDR, and ABCB1 play important roles in the pathogenesis and treatment of UC. STAT3, a member of the Stat protein family, plays a crucial role in cytokine signaling pathways (Hillmer et al., 2016). Various cytokines, such as IL-6 family cytokines, IL-10, IL-22, IL-17, and IL-23, can activate STAT3, leading to its phosphorylation and regulatory functions. In UC, STAT3 exhibits a dual role: on one hand, it contributes to the exacerbation of inflammation by inducing the expression of anti-apoptotic genes such as Bcl-2 and Bcl-xL in mucosal T cells through IL-6 activation, and on the other hand, its activation in intestinal epithelial cells contributes to UC treatment by maintaining intestinal homeostasis (Han and Theiss, 2014). Moreover, studies have suggested that IL-22, through STAT3 activation, protects intestinal epithelial cells from damage and promotes intestinal mucosal tissue repair (Yu et al., 2013). The *ABCB1* gene, formerly known as *MDR1*, encodes the P-glycoprotein (P-gp), one of the most extensively studied human ATP-binding cassette transporters. P-gp utilizes the energy generated by ATP hydrolysis to catalyze substrate transport against concentration gradients, preventing the absorption of drugs and toxins into capillaries and limiting the bioavailability of many orally administered drugs (Leschziner et al., 2007). It is known that ABCB1 knockout mice (MDR1a−/−) spontaneously develop colitis with pathological phenotypes similar to human UC (Resta-Lenert et al., 2005). Additionally, another study suggested that P-gp expression in the colon of Crohn’s disease patients is relatively reduced, and the expression of P-glycoprotein in intestinal epithelial cells contributes to maintaining the barrier function of the intestinal mucosa (Wilson et al., 2019). The *KDR* gene encodes the Vascular endothelial growth factor receptor 2 (VEGFR2) protein, which primarily participates in angiogenesis (Wang et al., 2020). Angiogenesis refers to the formation of new blood vessels based on the existing vascular network. Increasing clinical and experimental evidence indicates that angiogenesis also plays a crucial role in IBD. Compared to healthy intestines, microvascular physiology, and function in the intestinal tissues of IBD undergo significant changes. Furthermore, receptor phosphorylation within cells leads to the activation of various downstream signaling pathways. Among them, the phosphatidylinositol 3-kinase (PI3K) signaling pathway has become one of the major signaling pathways for VEGFR2-stimulated endothelial cell survival and proliferation (Alkim et al., 2015). Our Real-time PCR results indicate that LPS-induced transcription of STAT3 and ABCB1 (ABCB1a and ABCB1b) can be inhibited by LanC.

Despite its contributions, our study has certain limitations. The identification of altered expression genes in UC in our study was derived from two RNA-seq datasets, GSE87466 and GSE222070. While these datasets provided valuable insights, relying solely on them may introduce certain limitations to the generalizability of our findings. To further validate and refine the results, the inclusion of additional UC-related datasets in future analyses would be beneficial and could enhance the robustness and precision of our conclusions. Secondly, while molecular docking validation showed the binding ability of LanC to STAT3, KDR, and ABCB1, and we further confirmed its inhibition of STAT3 and ABCB1 transcription at the cellular level, but we did not conclusively demonstrate its effects on the biological activity of these proteins. Additionally, our investigation primarily focused on *in vitro* experiments, specifically evaluating LanC’s ability to suppress the production of inflammatory cytokines in a murine macrophage cell line. Given the substantial differences between *in vitro* and *in vivo* systems, further research is needed to validate LanC’s therapeutic potential for UC and elucidate its mechanisms of action. This would include assessing its safety *in vivo* and employing acute colitis models, such as DSS-induced colitis or TNBS-induced acute intestinal inflammation, to confirm its efficacy. Moreover, our research focused on a few core targets, while the UC pathogenesis may involve a broader range of molecules and pathways. Our findings should be viewed as a starting point, providing a theoretical basis for future investigations aimed at optimizing UC treatment strategies. To fully substantiate LanC’s therapeutic potential and elucidate its mechanisms, more comprehensive experimental validation and clinical studies will be required.

## 5 Conclusion

In this study, we employed network pharmacology to preliminarily explore the comprehensive overview of the therapeutic potential of LanC in UC. Our findings reveal that the therapeutic mechanisms of LanC are multifaceted, involving numerous protein targets, including STAT3, ABCB1, and VEGFR2, as well as diverse signaling pathways and cellular processes, such as “fatty acid metabolic process,” “xenobiotic metabolism,” and “cell metabolism.” To further elucidate its mechanisms of action, we conducted *in vitro* cellular experiments, which suggested that LanC’s pharmacological effects may be linked to its ability to inhibit the production of pro-inflammatory cytokines, including IL-1β, IL-6, and TNF-α. These findings provide a theoretical basis for advancing UC clinical treatment strategies and highlight LanC’s potential as a promising novel therapeutic option for UC.

## Data Availability

The datasets presented in this study can be found in online repositories. The names of the repository/repositories and accession number(s) can be found in the article/Supplementary Material.

## Supplementary material

SUPPLEMENTARY TABLE S1 The top 50 differentially expressed genes in UC from GSE87466 dataset.

SUPPLEMENTARY TABLE S2 The top 50 differentially expressed genes in UC from GSE222070 dataset.
