# Supplementary material for: Network pharmacology analysis of Lanatoside C: molecular targets and mechanisms in the treatment of ulcerative colitis
Source: Front Mol Biosci. 2025 Mar 21;12:1552360. doi: 10.3389/fmolb.2025.1552360 (PMC11968694; doi:10.3389/fmolb.2025.1552360)
Supplement: Supplementary file 1 [file Table1.docx]

| Gene name | LogFC | *P* value | Regulation direction |
| --- | --- | --- | --- |
| CHI3L1 | 5.716696 | 5.07E-35 | Up |
| SLC6A14 | 5.478665 | 1.26E-61 | Up |
| MMP3 | 5.44909 | 1.50E-29 | Up |
| DUOX2 | 5.375534 | 7.89E-54 | Up |
| DEFB4A | 5.087148 | 9.46E-21 | Up |
| CHI3L1.1 | 4.978753 | 4.75E-34 | Up |
| REG3A | 4.528524 | 3.05E-21 | Up |
| S100A8 | 4.508964 | 1.52E-23 | Up |
| SAA1.....SAA2 | 4.409415 | 5.46E-25 | Up |
| REG1A | 4.404567 | 2.76E-17 | Up |
| S100A8.1 | 4.219181 | 1.75E-22 | Up |
| CXCL1 | 4.147422 | 2.29E-42 | Up |
| REG1B | 4.142779 | 3.20E-11 | Up |
| IL8 | 4.124758 | 3.38E-21 | Up |
| TNIP3 | 4.103176 | 3.86E-26 | Up |
| SAA1.....SAA2.1 | 4.022237 | 2.64E-30 | Up |
| X.5234 | 3.816046 | 1.50E-24 | Up |
| DEFA5 | 3.704609 | 2.52E-13 | Up |
| VNN1.1 | 3.588066 | 1.54E-25 | Up |
| MMP10 | 3.581145 | 2.47E-24 | Up |
| DEFA6 | 3.469109 | 2.98E-12 | Up |
| KLK10 | 3.453876 | 2.64E-11 | Up |
| FCGR3B | 3.43702 | 3.89E-15 | Up |
| SERPINB5.1 | 3.42922 | 1.22E-18 | Up |
| DUOXA2 | 3.383836 | 6.55E-25 | Up |
| TCN1 | 3.349205 | 9.61E-20 | Up |
| AQP9 | 3.333624 | 9.67E-12 | Up |
| CXCL2.1 | 3.281056 | 1.24E-32 | Up |
| GPR109B | 3.263685 | 6.07E-14 | Up |
| PROK2 | 3.253528 | 9.73E-10 | Up |
| LOC100288985 | 3.253383 | 5.91E-16 | Up |
| CXCL9 | 3.199963 | 1.12E-26 | Up |
| SOCS3.2 | 3.187256 | 3.69E-29 | Up |
| IDO1 | 3.187071 | 3.47E-23 | Up |
| AQP8 | -5.09127 | 1.08E-28 | Down |
| OSTalpha | -4.18577 | 1.11E-31 | Down |
| SLC26A2 | -4.00271 | 1.27E-18 | Down |
| HMGCS2.1 | -3.92939 | 2.25E-34 | Down |
| CLDN8 | -3.82903 | 3.25E-08 | Down |
| PCK1 | -3.74382 | 5.27E-13 | Down |
| SLC26A2.2 | -3.67205 | 2.08E-16 | Down |
| LOC389023 | -3.6708 | 1.19E-55 | Down |
| SLC38A4.1 | -3.62529 | 1.04E-81 | Down |
| HMGCS2 | -3.39064 | 1.19E-16 | Down |
| GBA3 | -3.31113 | 5.47E-22 | Down |
| ABCG2 | -3.27589 | 4.57E-21 | Down |
| X.8632 | -3.26146 | 2.18E-12 | Down |
| MEP1B | -3.24024 | 4.66E-30 | Down |
| CYP2B6 | -3.22938 | 5.84E-24 | Down |
| GBA3.1 | -3.21235 | 2.47E-24 | Down |

**Supplementary Table 1. The top 50 differentially expressed genes in UC from GSE87466 dataset.**
