# Supplementary material for: Network pharmacology analysis of Lanatoside C: molecular targets and mechanisms in the treatment of ulcerative colitis
Source: Front Mol Biosci. 2025 Mar 21;12:1552360. doi: 10.3389/fmolb.2025.1552360 (PMC11968694; doi:10.3389/fmolb.2025.1552360)
Supplement: Supplementary file 2 [file Table2.docx]

| Gene name | LogFC | *P* value | Regulation direction |
| --- | --- | --- | --- |
| FLJ40194 | 5.070039 | 5.35E-08 | Up |
| PRAC | 4.864031 | 1.84E-06 | Up |
| DCDC5 | 4.862981 | 1.37E-05 | Up |
| C9orf171 | 4.821917 | 3.91E-07 | Up |
| SLC22A25 | 4.655223 | 1.77E-05 | Up |
| FAM205A | 4.395175 | 0.000626 | Up |
| CDRT15L2 | 4.31448 | 8.31E-05 | Up |
| TMEM174 | 4.291357 | 0.00016 | Up |
| STAR | 4.277447 | 0.001467 | Up |
| C20orf26 | 4.24359 | 5.28E-07 | Up |
| FAM26D | 4.218896 | 0.001216 | Up |
| OR5J2 | 4.21823 | 6.13E-05 | Up |
| WDR93 | 4.169007 | 0.000233 | Up |
| GJD4 | 4.092115 | 3.16E-07 | Up |
| IGFL3 | 4.050374 | 5.45E-08 | Up |
| POTED | 4.04795 | 6.32E-05 | Up |
| LCE3E | 3.97683 | 0.000111 | Up |
| MAGEA2B | 3.973378 | 3.64E-09 | Up |
| DEFB124 | 3.91925 | 1.39E-05 | Up |
| GAGE2A | 3.885636 | 0.008613 | Up |
| MAGEA2 | 3.849513 | 4.33E-09 | Up |
| LOC100128338 | 3.807719 | 1.19E-05 | Up |
| AQP7P1 | 3.766457 | 0.00022 | Up |
| SPATA31C1 | 3.763838 | 0.002718 | Up |
| NPVF | 3.751844 | 2.37E-07 | Up |
| RSPH6A | 3.737123 | 9.11E-07 | Up |
| CXorf48 | 3.682592 | 6.57E-05 | Up |
| OR5D14 | 3.675809 | 0.00011 | Up |
| IL22 | 3.668748 | 0.000296 | Up |
| OTX2 | 3.649059 | 0.000108 | Up |
| RGS4 | 3.569418 | 1.66E-05 | Up |
| KIAA0408 | 3.55577 | 2.05E-12 | Up |
| OR10H1 | 3.544777 | 0.000542 | Up |
| OR13D1 | 3.530987 | 2.31E-05 | Up |
| OCM2 | 3.511033 | 2.85E-06 | Up |
| SFTPA1 | 3.506992 | 0.002592 | Up |
| SAA1 | 3.460288 | 4.03E-05 | Up |
| SLC51A | -4.53005 | 3.27E-06 | Down |
| ALPI | -4.52729 | 0.000134 | Down |
| SLC6A19 | -4.39766 | 5.70E-05 | Down |
| PRAP1 | -4.36994 | 0.000649 | Down |
| GBA3 | -4.21637 | 0.000529 | Down |
| RBP2 | -4.19009 | 1.01E-05 | Down |
| SSTR1 | -3.97719 | 2.53E-05 | Down |
| CYP4F2 | -3.94466 | 0.000171 | Down |
| TMIGD1 | -3.76791 | 0.015353 | Down |
| HRSP12 | -3.60406 | 0.011413 | Down |
| MEP1B | -3.54388 | 0.00234 | Down |
| BCMO1 | -3.52135 | 0.000438 | Down |
| BMP3 | -3.51448 | 0.014633 | Down |

**Supplementary Table 2. The top 50 differentially expressed genes in UC from GSE222070 dataset**.
